# Mapping the EQ-5D index from the cystic fibrosis questionnaire-revised using multiple modelling approaches

**DOI:** 10.1186/s12955-015-0224-6

**Published:** 2015-03-12

**Authors:** Sarah Acaster, Binny Pinder, Clara Mukuria, Amanda Copans

**Affiliations:** Oxford Outcomes Ltd, an Icon plc Company, Seacourt Tower, West Way, Oxford, OX2 0JJ UK; ScHARR, University of Sheffield, Regent Court, 30 Regent Street, Sheffield, S1 4DA UK; Gilead Sciences Inc, 333 Lakeside Drive, Foster City, CA 94404 USA

**Keywords:** Mapping, Health utilities, CFQ-R, EQ-5D, Quality of life

## Abstract

**Background:**

This study was designed to develop a mapping algorithm to estimate EQ-5D utility values from Cystic Fibrosis Questionnaire-Revised (CFQ-R) data.

**Methods:**

A cross-sectional survey of adults with cystic fibrosis (CF) was conducted in the UK. The survey consisted of the CFQ-R, the EQ-5D and a background questionnaire. Eight regression models, exploring item and domain level predictors, were evaluated using three different modelling approaches: ordinary least squares (OLS), Tobit, and a two-part model (TPM). Predictive performance in each model was assessed by intraclass correlations, information criteria (Bayesian information criteria and Alkaike information criteria), and root mean square error (RMSE).

**Results:**

The survey was completed by 401 participants. For all modelling approaches the best performing item level model included all items, and the best performing domain level model included the CFQ-R Physical-, Role- and Emotional-functioning, Vitality, Eating Disturbances, Weight, and Digestive Symptoms domains and a selection of squared terms. Overall, the item level TPM, including age and gender covariates performed best within sample validation, but OLS and TPM domain models with squared terms performed best out-of-sample and are recommended for mapping purposes.

**Conclusions:**

Domain and item level models using all three modelling approaches reached an acceptable degree of predictive performance with domain models performing well in out-of-sample validation. These mapping functions can be applied to CFQ-R datasets to estimate EQ-5D utility values for economic evaluations of interventions for patients with cystic fibrosis. Further research evaluating model performance in an independent sample is encouraged.

**Electronic supplementary material:**

The online version of this article (doi:10.1186/s12955-015-0224-6) contains supplementary material, which is available to authorized users.

## Background

Cystic fibrosis (CF) is a hereditary and life-threatening autosomal recessive disorder. An estimated 80,000 children and young adults suffer with CF worldwide, with a rate of 1 case per 2,500 births [1]. If untreated, patients are likely to suffer from chronic respiratory infections, pancreatic enzyme insufficiency and associated complications. Advances in treatment and management have resulted in an increase in survival rates. The predicted median age of survival for a person with CF is the late 30s, and with over half of children born in the 1990s expected to survive into their fifth decade [2]. Despite these advances though the disease still represents a very significant burden for patients in terms of their symptoms, loss of functioning and poor health related quality of life (HRQL) [3].

HRQL is a multi-dimensional concept, which reflects individual’s subjective evaluation of his or her daily functioning (i.e. physical, psychological, emotional and social functioning) and well-being. Poor lung functioning [Forced Expiratory Volume in 1 second (FEV_1_) < 30% predicted] and pulmonary exacerbations in the past 6 months have been related to poor HRQL [4,5]. The Cystic Fibrosis Questionnaire-Revised (CFQ-R) is a validated patient-reported outcome (PRO) measure of HRQL specifically designed for individuals with CF [6,7]. The CFQ-R is commonly used in CF clinical trials where it has demonstrated responsiveness [8,9], and been used to support PRO label claims.

Decision makers within drug licensing authorities such as the US Food and Drug Administration (FDA) and payers such as the National Institute for Health & Care Excellence (NICE) in the UK have become increasingly interested in the information that can be captured from HRQL PROs. NICE and many other health technology assessment bodies globally, are interested in understanding the benefits of health technologies in terms of quality-adjusted life years (QALYs): a metric incorporating length and quality of life. Estimating QALY requires a specific type of HRQL data that reflects the value that people place on HRQL rather than just a psychometric score. This value is referred to as utility and is measured on a scale of 0 (dead) to 1 (full health). UK national guidelines regarding the data used in health technology appraisals recommend the use of generic preference-based measures to capture utility, with a stated preference for the EQ-5D questionnaire [10]. However these data are not always collected in clinical trials. To address this data gap it is possible to estimate EQ-5D scores from a different PRO, such as the CFQ-R, with the development of a robust mapping algorithm. Mapping studies often also incorporate demographic characteristics into model estimation to increase a models predictive performance [11-13]. This approach is endorsed by NICE [14] and there is a growing body of literature related to the development of mapping functions linking *source* disease specific HRQL measures onto *target* preference-based measured using regression models [15].

The present study was designed to develop a mapping algorithm to estimate EQ-5D utility values from CFQ-R data, with and without adjustment for demographic characteristics (age and gender). This will enable existing and future trial datasets, which include CFQ-R (but not EQ-5D), to be used by decision makers to understand the value of new health technologies in CF.

## Materials and methods

### Study design and participants

A cross-sectional observational study conducted as an on-line survey was undertaken in the UK. The option to complete a pen and paper survey through the post was provided but not utilised by any respondents. The survey was advertised by the Cystic Fibrosis Trust (CF Trust) by placing adverts on the CF Trust website, forum, Facebook page, Twitter account and Google Adword. Potential respondents were informed that the CF Trust would receive a £50 donation for every completed survey; respondents did not receive any direct remuneration for their participation.

All participants had a self-reported clinical diagnosis of CF, were aged 18 years or above and currently resident in the UK. Participants were also asked to rate their CF severity as mild, moderate or severe during screening to ensure sample variability in HRQL item responses.

### Ethics

Independent ethical review was sought and granted by Schulman Associates Independent Institutional Review Board Inc. Informed consent was obtained from all participants prior to completion of the online survey.

### Survey

Interested participants followed a link provided by the CF Trust to be taken to an information sheet describing the purpose of the survey, the consent form and the survey. The survey was conducted from January – March 2012. The survey consisted of three questionnaires: the CFQ-R, the EQ-5D, and a demographic/clinical background form. Each of these measures is descried in more detail below.

### CFQ-R

The CFQ-R is a validated disease-specific questionnaire measuring health-related quality of life in CF patients [6,7]. The teen/adult UK English version of the questionnaire, suitable for ages 14+, was used. This consists of 50 items across 12 domains: ‘physical functioning’, ‘role functioning’, ‘emotional functioning’, ‘vitality’, ‘social functioning’, ‘body image’, ‘eating disturbances’, ‘treatment burden’, ‘health perceptions’, ‘weight’, ‘respiratory symptoms’, and ‘digestive symptoms’. All items use categorical response options, with values ranging from 1 – 4. Domain scores were calculated using the developer’s guidelines, which produces a potential range of scores from 0–100, with higher scores indicting better HRQL.

### EQ-5D

The EQ-5D-3L is a generic preference-based measure of HRQL [16-18]. The questionnaire consists of five domains: ‘mobility’, ‘self-care’, ‘usual activity’, ‘pain/discomfort’, and ‘anxiety/depression’. Participants also indicate their current health on a visual analogue scale ranging from 0 (worst imaginable health state) to 100 (best imaginable health state). Health utilities were derived from the EQ-5D using UK general population preference weights [19], which provide a potential range of scores from - 0.59 to 1.0; a score of 1 represents full health, a score of 0 represents a state equivalent to dead, and a score below 0 represents a state worse than dead. NICE state that EQ-5D is the preferred source of utility values for use in economic evaluation [10].

### Demographic/clinical background form

The demographic/clinical background form gathered data on respondents’ age, sex, ethnicity, employment status, time since CF diagnosis, FEV_1_ (if known), date of last FEV_1_ assessment, and exacerbation occurrence since last FEV_1_ assessment.

None of the respondents had missing data in the EQ-5D, CFQ-R, age or gender.

### Analysis

#### Model development and specifications

**Figure 1:**
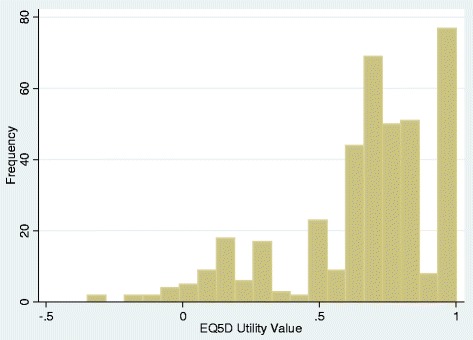
**Histogram of EQ-5D utility scores.**

Figure 1 shows the distribution of EQ-5D utility scores, which was used to determine which modelling approaches to use. 19% had a score of 1 (i.e. full heath) while 3% had a score less than 0. Three regression modelling approaches were used to identify the most parsimonious prediction model with the best fit: an ordinary least squares (OLS) model, a Tobit model, and a two-part model (TPM). The OLS approach is used to estimate the unknown parameters in a linear regression model by minimizing the sum of squared errors from the data. This model has frequently been identified as the most parsimonious and best fitting model in utility mapping studies when compared to other methods designed to cope with bounded and multi-modal distributions [15,20]. The Tobit model (also known as the censored regression model) takes better account of the censored nature of EQ-5D data, deals with truncated data and can approximate for skewed data by setting the upper limit to 1. Censored least absolute deviation (CLAD) models have also been advocated to deal with censoring but these are median-based models while most economic evaluation models are mean-based [14] therefore CLAD was not assessed. The TPM approach deals with the high proportion of values are at 1.0 [21-23]. The first part of the two-part model uses a logit regression to estimate the probability that an individual is in full health. The second part estimates EQ-5D utilities for remaining observations using a truncated OLS model which can lie between −0.594 and 0.99. The two parts of the model are combined using the expected value method to calculate the EQ-5D score as:

$$ EV\ \left(EQ-5D\right) = \left[P\left(EQ-5D=1\right)\ *\ 1\right] + \left[P\left(EQ-5D\ne 1\right)\ *\  Predicted\ EQ-5D\  part\ 2\right] $$

[EV = Expected value; P(EQ-5D = 1) = Probability of being at score 1 predicted from part 1; (P(EQ-5D ≠ 1) = 1- P(EQ-5D = 1), probability of not being at 1; Predicted EQ-5D part 2 = predicted EQ-5D from a truncated OLS regression for those who score less than 1].

Based on recommendations in the literature [14], separate models were tested for the CFQ-R domains and items were used to predict EQ-5D utility scores using the three modelling approaches. The CFQ-R Health domain and its constituent items were not selected for the regression models as all of the remaining CFQ-R items also measured health; thus these items would either be redundant or cause problems of multicollinearity which would violate the regression assumptions and render the model unreliable. Item 43 (*How has your mucus been?*) from the CFQ-R was also removed from the regression analysis as this item was a sub-question which not all participants provided a response. Gender and age were also included in one of the regression specifications for all three models. Self reported FEV_1_ was not included in any models as the aim was to estimate a mapping function specifically from the CFQ-R, rather than a combination of measures of CF. In total, eight different sets of independent variables were evaluated to ensure the best model specification was selected and repeated using OLS, Tobit and TPM mapping methods:

- Model 1: All CFQ-R domains excluding the health domain
- Model 2: CFQ-R domains that are statistically significant at the 10% level
- Model 3: Model 2 + statistically significant squared terms
- Model 4: Model 3 + interaction terms
- Model 5: All CFQ-R items excluding the health domain items
- Model 6: CFQ-R items that are statistically significant at the 10% level
- Model 7: Model 6 with collapsed unordered items
- Model 8: Best fitting model + gender and age

In all item level models, the items were reverse coded if appropriate, and dummy coded with a score of 1 (poor health) as the reference category. In Model 7 unordered items (where coefficients did not follow the predicted order of magnitude across good to poor response options) were dichotomised to ‘no problems’ versus ‘other’. In the TPM model, item level models were also collapsed to 2 or 3 levels for those in full health.

The Ramsey Regression Equation Specification Error Test was used to assess misspecification in the linear models obtained using OLS. The linktest was used to assess misspecification in the Tobit model and the second part of the TPMs. Multicollinearity was assessed using the variance inflation factor (VIF) with values greater than 10 indicating a problem. Bootstrapped bias-corrected (2000 replications) or robust standard errors are reported for all models.

### Model validation and comparison

Model goodness of fit was assessed by adjusted/pseudo R^2^ statistics (OLS and Tobit models only), Bayesian information criteria (BIC) and Alkaike information criteria (AIC) statistics. Lower BIC and AIC values would indicate a better fitting model. To examine the predictive performance of the model the differences between the predicted and observed EQ-5D scores at the individual level were examined by computing the mean squared error (MSE) and root MSE (RMSE). Smaller error values are indicative of better performing models. Plots of the observed and predicted EQ-5D scores are used to examine the performance of the models. Predicted and observed EQ-5D utility scores and RMSE were also compared across different EQ-5D ranges and CF severity as measured by percentage of predicted FEV_1_ (FEV_1_ groups: mild = >70%, moderate = 70% - 41%, severe = < 41%). ANOVA models were used to examine differences in predicted scores across EQ-5D ranges and FEV_1_ severity groups. Intra-class correlations, which measure the level of agreement between the predicted and observed scores, were also assessed.

It is recommended that where possible an external dataset is used as a validation dataset to determine the accuracy of predicted utility values of the selected models out-of-sample [14]. However, no external dataset was available for the present study and therefore the performance of the mapping algorithms were assessed using a cross-validation approach. The sample was randomly split into four groups of 25% each. The best fitting models within sample were re-run on three of the four group and applied to the excluded group to ensure in an iterative process until each of the samples had been used as both estimation and validation samples. 75% of the data were used as an estimation dataset for building models, and 25% were used as a validation dataset. The proportion of responses for the estimation dataset is larger than for the validation dataset to enhance model accuracy with a greater number of responses.

All regression analyses were conducted using STATA v 11.

## Results

### Sample characteristics

A total of 401 participants completed the survey; all surveys were completed online. The demographic and clinical characteristics of participants, by FEV_1_ severity group and for the sample as a whole, are presented in Table 1. The sample represented a broad range in terms of demographics and disease severity.

**Table 1:** **Demographic and clinical characteristics of the study participants**

**Characteristic**		**Statistic**
**N**		401
**Age**	Mean ± SD	28.7 ± 8.88
	Range (min, max)	18 - 62
**Sex**	Male: N (%)	156 (38.9)
**Ethnicity (N %)**	White	393 (98.0)
	Other	8 (2.0)
**Working/studying**	Yes: N (%)	248 (49.4)
**Diagnosis of CF (years)**	Mean ± SD	25.3 ± 9.1
	Median	25.0
	Range (min, max)	1 - 59
**Recent FEV**_**1**_ **Predicted**	Mean ± SD	65.7 ± 27.3
	Median	67.0
	Range (min, max)	17 - 99
**Last FEV**_**1**_ **: N (%)**	0 – 1 month	242 (63.9)
	>1 - 3 months	97 (25.6)
	>3 months	11 (2.9)
**Recent Exacerbation: N (%)**	Yes	114 (30.1)
**Requiring Hospitalisation**	N (%) of above	42 (36.8)

N, sample size; SD, Standard Deviation; FEV_1_, percentage of predicted Forced Expiratory Volume in 1 second.

FEV_1_ severity levels: Mild = >70%; Moderate = 41% – 70%; Severe = < 41%.

### Descriptive statistics for EQ-5D and CFQ-R

Observed EQ-5D utility and CFQ-R domain scores of the participants are shown in Table 2. The mean EQ-5D score was 0.67 (SD = 0.28), ranging from – 0.35 to 1, which is only slightly narrower than the theoretical range of – 0.59 to 1. Both the EQ-5D and CFQ-R mean scores reflect the self-reported disease severity as measured by FEV_1_, with utility and almost all CFQ-R domain scores declining with increased severity. The digestive symptoms domain was the only domain not reflecting FEV_1_ severity.

**Table 2:** **EQ-5D and CFQ-R descriptive data - total sample and split by FEV**_**1**_ **severity levels**

	**Mild FEV**_**1**_	**Moderate FEV**_**1**_	**Severe FEV**_**1**_	**Total sample**
	**Mean ± SD**	**Mean ± SD**	**Mean ± SD**	**Mean ± SD**
***EQ-5D***				
**Utility value**	0.74 ± 0.27	0.70 ± 0.26	0.54 ± 0.29	0.67 ± 0.28
***CFQ-R***				
**Physical functioning**	67.18 ± 28.82	44.74 ± 26.01	20.63 ± 18.24	45.71 ± 30.83
**Role functioning**	71.83 ± 25.25	60.17 ± 24.00	41.87 ± 26.19	59.87 ± 27.54
**Vitality**	47.78 ± 23.06	41.09 ± 20.78	31.73 ± 17.68	40.69 ± 21.74
**Emotional functioning**	62.54 ± 25.49	57.89 ± 22.47	49.64 ± 19.82	57.47 ± 23.44
**Social functioning**	61.06 ±17.84	55.06 ± 19.26	50.94 ± 20.51	56.12 ± 19.35
**Body image**	64.23 ± 28.07	61.84 ± 27.27	43.91 ± 29.75	58.38 ± 28.96
**Eating disturbance**	77.35 ± 25.22	76.32 ± 25.33	65.73 ± 28.89	74.70 ± 26.28
**Treatment burden**	56.30 ± 23.68	44.83 ± 23.87	42.17 ± 22.61	49.40 ± 25.2
**Health perceptions**	58.94 ± 26.43	43.45 ± 21.23	24.90 ± 22.66	44.25 ± 26.16
**Weight**	69.52 ± 36.43	63.91 ± 39.77	42.17 ± 40.35	60.76 ± 40.29
**Respiratory symptoms**	61.00 ± 24.68	47.82 ± 21.48	38.55 ± 21.8	49.63 ± 24.44
**Digestive symptoms**	67.30 ± 24.65	71.80 ± 25.63	73.49 ± 22.00	71.27 ± 23.92

SD, standard deviation; FEV_1_, percentage of predicted Forced Expiratory Volume in 1 second.

FEV_1_ severity levels: Mild = >70%; Moderate = 41% – 70%; Severe = < 41%.

### Regression modelling

24 models were explored in total (8 specifications for OLS, Tobit and TPM), the goodness of fit and predictive performance statistics from the best domain and item level model for each regression type are presented in Table 3. The identification of the best domain and item level models was based on an examination of all goodness of fit and predictive performance statistics. The performance statistics of the 18 models not presented are available upon request.

**Table 3:** **Summary of model performance for best OLS Tobit and TPM Item and domain models**

	**Observed EQ-5D**	**OLS models**		**Tobit models**		**TPM models**	
		**Domain (3)**	**Item (5)**	**Domain (3)**	**Item (5)**	**Domain (3)**	**Item (8)**
**Mean**	0.671	0.671	0.671	0.672	0.672	0.691	0.679
**SD**	0.282	0.223	0.245	0.225	0.250	0.236	0.254
**Range of values**	- 0.349 - 1	- 0.099 - 1.04	- 0.165 - 1.183	- 0.053 - 0.985	- 0.167 - 1	- 0.221 - 0.985	- 0.200 - 1
**ICC**		0.715	0.801	0.716	0.811	0.717	0.820
**RMSE**	-	0.127	0.111	0.173	0.136	0.127	0.096
**MSE**	-	0.030	0.029	0.030	0.018	0.029	0.017
**BIC**	-	−214	383	45	626	256, − 201	574, 379
**AIC**	-	−254	−173	5	71	240, − 247	270, − 150

OLS, ordinary least squares; TPM, two-part model; SD, standard deviation; Adj. R^2^, adjusted R^2^; RMSE, root of the mean square error; MSE, mean square error; AIC, Akaike information criterion; BIC, Bayesian information criterion.

Model 3 = CFQ-R domains that are statistically significant at the 10% level + statistically significant squared terms; Model 5 = All CFQ-R items excluding the health domain items; TPM Model 8 = All CFQ-R items excluding the health domain items + age and gender.

In the OLS, Tobit and TPM regressions, the best performing domain level model within sample was model 3: including statistically significant domains at the 10% level, plus significant squared terms. There was no evidence of multicollinearity in any of the domain level models (mean and individual variable VIF < 10) apart from where expected when squared terms are included. There was evidence of misspecification in all the OLS models including model 3 but the Tobit and TPM model 3 were not misspecified. The best performing item level model within sample was model 5 (all CFQ-R items included in analysis) for OLS, Tobit and TPM; however, the TPM model 5 was improved with the addition of age and gender as covariates (model 8). Item level models had mean VIF <10 but some individual dummy variables (19/139) had VIF greater than 10 which indicates problems with multicollinearity when all items were included. Item level models also had evidence of misspecification for all models apart from model 7.

**Figure 2:**
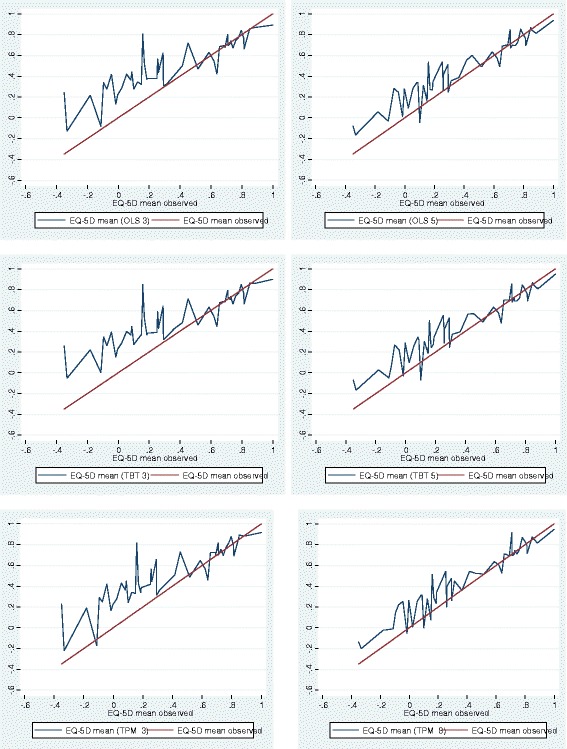
**Observed and Predicted EQ-5D for Best Fitting Item and Domain Models for OLS, Tobit and TPM.**

As shown in Table 3 all six best performing models demonstrated good predictive performance within sample; all predicted means (0.671 – 0.691) were within 0 – 0.02 of the observed mean (0.671), and the fitted ranges of the EQ-5D preference-based values were within 0.128 – 0.296 of the lower bound observed value (−0.349). As would be expected only OLS models exceeded the upper bound observed value of 1. In all instances the item level models performed marginally better than the domain level models. All the models showed good ICC (>0.7) between predicted and observed EQ-5D values. This is further illustrated in Figure 2 and Table 4, where the mean observed and predicted EQ-5D preference-based values by health state ranking indicate over prediction for more severe health states (where the observed EQ-5D value was less than 0.3), and under prediction for very mild health states (where the observed EQ-5D value was above 0.9). However, Table 4 also illustrates that all six best performing domain and item level models demonstrated responsiveness to severity as assessed by EQ-5D and FEV_1_ sub-groups. There were statistically significant differences (all p’s < 0.001) across EQ-5D and FEV_1_ health states for each model’s predicted EQ-5D values. The best performing within sample model overall was the item level TPM, including age and gender covariates. This model performed best when predicting values across the range of EQ-5D observed scores, did not include out of range predicted values, and demonstrated good predictive performance with the lowest RMSE values.

**Table 4:** **Summary of observed and predicted values by EQ-5D group and FEV**_**1**_ **severity**

**Observed EQ-5D range**	**N**	**Observed EQ-5D**	**OLS models**		**Tobit models**		**TPM models**	
			**Domain (3)**	**Item (5)**	**Domain (3)**	**Item (5)**	**Domain (3)**	**Item (8)**
**−0.349 - 0.099**	23	−0.028	0.294 (0.321)	0.197 (0.225)	0.293 (0.320)	0.185 (0.214)	0.274 (0.307)	0.160 (0.196)
**0.1 - 0.299**	41	0.227	0.420 (0.211)	0.337 (0.149)	0.421 (0.209)	0.335 (0.148)	0.418 (0.214)	0.320 (0.134)
**0.3 - 0.599**	38	0.511	0.505 (0.130)	0.518 (0.106)	0.502 (0.136)	0.517 (0.113)	0.524 (0.135)	0.532 (0.100)
**0.6 - 0.699**	92	0.660	0.620 (0.106)	0.643 (0.093)	0.621 (0.110)	0.643 (0.098)	0.649 (0.115)	0.655 (0.092)
**0.7 - 0.799**	71	0.756	0.747 (0.084)	0.736 (0.088)	0.749 (0.093)	0.737 (0.097)	0.778 (0.099)	0.761 (0.089)
**0.8 - 0.899**	59	0.841	0.794 (0.109)	0.804 (0.097)	0.792 (0.103)	0.804 (0.100)	0.817 (0.101)	0.816 (0.099)
**0.9 - 1**	77	1	0.895 (0.107)	0.938 (0.088)	0.900 (0.100)	0.949 (0.051)	0.916 (0.084)	0.948 (0.052)
ANOVA			F = 107.30	F = 176.10	F = 105.70	F = 183.84	F =104.81	F = 202.73
			*p* < 0.001	*p* < 0.001	*p* < 0.001	*p* < 0.001	*p* < 0.001	*p* < 0.001
**FEV**_**1**_								
**Severe < 41%**	92	0.552	0.550 (0.157)	0.548 (0.132)	0.548 (0.158)	0.550 (0.133)	0.565 (0.157)	0.561 (0.121)
**Moderate 41-70%**	136	0.695	0.684 (0.119)	0.694 (0.107)	0.683 (0.120)	0.693 (0.101)	0.703 (0.120)	0.702 (0.099)
**Mild > 70%**	105	0.741	0.755 (0.113)	0.748 (0.097)	0.760 (0.116)	0.751 (0.090)	0.781 (0.111)	0.757 (0.076)
ANOVA			F = 24.38	F = 19.29	25.39	F = 18.43	F = 23.83	F = 16.76
			*p*< 0.001	*p*< 0.001	*p* < 0.001	*p* < 0.001	*p* < 0.001	*p* < 0.001

OLS, ordinary least squares; TPM, two-part model; FEV_1_, percentage of predicted Forced Expiratory Volume in 1 second.

Model 3 = CFQ-R domains that are statistically significant at the 10% level + statistically significant squared terms; Model 5 = All CFQ-R items excluding the health domain items; TPM Model 8 = All CFQ-R items excluding the health domain items + age and gender.

All 6 models were tested in the out-of-sample cross-validation. A one way analysis of variance test indicated no significant differences between the mean observed EQ-5D values of the validation and estimation samples across the 4 samples (F_397,3_ = 0.05, p = 0.985). Table 5 provides summary statistics of the observed and predicted EQ-5D utility scores in each of the four samples based on models ran in the other 3 samples e.g. sample 1 predicted scores are based on models undertaken in the combined 2 to 4 samples. Mean values tend to be larger or smaller (difference 0.001 to 0.02) than the observed mean values for most of the models with either OLS and Tobit domain models (model 3) having the smallest differences in samples 1 to 3 and TPM item model (Model 8) having the smallest difference in sample 4. In all the samples apart from sample 2, all the models perform poorly at predicting the full observed range particularly at the poor end of health (difference 0.03 to 0.62). In sample 2 the OLS item model (model 5) and the TPM domain model (model 3) are within 0.004 of the observed minimum score. Tobit and TPM item models (5 and 8) predict the maximum accurately while OLS models predict values greater than 1 particularly in the item models. In all the samples, RMSE is smallest in the OLS and TPM domain models (0.118 to 0.146) and largest in the TPM item level models (0.182 to 0.223). ICC is larger in the domain models (0.50 to 0.81) compared to the item models (0.29 to 0.56) indicating better agreement between observed and predicted scores in the former. Assessment of RMSE across the EQ-5D range indicates that all models are poor at predicting at the poor end of health but TPM item level models also have larger RMSE in other parts of the EQ-5D range as well (see the Additional file 1 detailing the results of Table 4 for each of the 4 cross-validation samples).

**Table 5:** **Out-of sample Cross validation of best fitting models - summary of observed and predicted values**

	**EQ-5D**	**OLS model 3**	**OLS model 5**	**Tobit model 3**	**Tobit model 5**	**TPM model 3**	**TPM model 5**
**Sample 1**							
**N**	97	97	97	97	97	97	97
**Mean (SD)**	0.6776 (0.277)	0.6755 (0.205)	0.6976 (0.252)	0.6717 (0.207)	0.6891 (0.238)	0.6932 (0.214)	0.6811 (0.270)
**Range**	−0.3490 - 1	0.2702 - 1.028	0.1038 - 1.269	0.2539 - 0.9729	0.1444 - 1	0.1814 - 0.9734	0.0133, 1
**RMSE**		0.141	0.163	0.160	0.160	0.187	0.187
**ICC**		0.65	0.54	0.65	0.53	0.64	0.40
**Sample 2**							
**N**	101	101	101	101	101	101	101
**Mean (SD)**	0.6632 (0.288)	0.6701 (0.243)	0.6744 (0.297)	0.6683 (0.246)	0.6744 (0.297)	0.6868 (0.263)	0.6785 (0.329)
**Range**	−0.1810 - 1	−0.0399 - 1.006	−0.1850 - 1.242	−0.0040 - 0.981	−0.2001 - 1	−0.1830 - 0.9838	−0.1642, 1
**RMSE**		0.118	0.165	0.162	0.162	0.182	0.182
**ICC**		0.79	0.61	0.79	0.64	0.79	0.50
**Sample 3**							
**N**	100	100	100	100	100	100	98
**Mean (SD)**	0.6742 (0.277)	0.6696 (0.202)	0.6791 (0.221)	0.6779 (0.210)	0.6808 (0.236)	0.6954 (0.214)	0.6886 (0.252)
**Range**	−0.1810 - 1	0.2417 - 0.9782	0.0777 - 1.193	0.2592 - 0.9824	−0.0555 - 1	0.2593 - 0.9837	0.1442, 1
**RMSE**		0.146	0.196	0.204	0.204	0.223	0.223
**ICC**		0.50	0.31	0.52	0.29	0.49	0.29
**Sample 4**							
**N**	103	103	103	103	103	103	103
**Mean (SD)**	0.6688 (0.287)	0.6703 (0.238)	0.6515 (0.281)	0.6703 (0.237)	0.6526 (0.302)	0.6887 (0.248)	0.6678 (0.295)
**Range**	−0.3310 - 1	−0.0577 - 1.048	−0.2275 - 1.201	−0.0441 - 0.9839	−0.2964 - 1	−0.1583 - 0.9879	−0.1745, 1
**RMSE**		0.120	0.185	0.180	0.180	0.207	0.207
**ICC**		0.80	0.51	0.81	0.56	0.81	0.47

OLS, ordinary least squares; TPM, two-part model; SD, standard deviation; ICC, Intraclass correlation; RMSE, root of the mean square error.

Model 3 = CFQ-R domains that are statistically significant at the 10% level + statistically significant squared terms; Model 5 = All CFQ-R items excluding the health domain items; TPM Model 8 = All CFQ-R items excluding the health domain items + age and gender.

Based on RMSE and ICC and mean predicted values, the OLS and TPM domain model (model 3) perform best out-of-sample but are not good at predicting the range of values. This contrasts with within sample predictions where TPM item model (model 8) performs best. This may be in part due to poor performance of these models when the samples are smaller as is the case when running the models in only 75% of the sample. However, the item models also have misspecification and multicollinearity, which may increase the variation in predicted scores. We therefore recommend the OLS or TPM model 3 (Table 6) for generating EQ-5D utility scores where they are not available.

**Table 6:** **OLS and TPM Model 3 coefficients**

	**OLS Model 3**				**TPM Model 3**							
					**Part 1**				**Part 2**			
**Variable**	**Coefficient**	**Bootstrapped SE**	**95% Bootstrapped SE (Bias-corrected)**				**95% Bootstrapped SE (Bias-corrected)**				**95% Bootstrapped SE (Bias-corrected)**	
**Physical**	0.00651***	(0.00141)	0.0037	0.0091	0.02836***	(0.00834)	0.0113	0.0449	0.00615***	(0.00188)	0.0027	0.0101
**Role**	0.00287***	(0.00057)	0.0017	0.0040	0.03285***	(0.01221)	0.0114	0.0583	0.00336***	(0.00075)	0.0019	0.0048
**Emotion**	0.00693***	(0.00211)	0.0028	0.0110	0.04287***	(0.01020)	0.0247	0.0637	0.00821***	(0.00277)	0.0029	0.0134
**Vitality**	0.00127**	(0.00062)	0.0001	0.0025					0.00592**	(0.00257)	0.0009	0.0112
**Eat**	0.00154***	(0.00053)	0.0005	0.0026					0.00206***	(0.00067)	0.0008	0.0034
**Weight**	−0.00058**	(0.00028)	−0.0011	−0.00003					−0.00090**	(0.00040)	−0.0017	−0.0001
**Digest**	0.00094**	(0.00044)	0.0001	0.0018					0.00106*	(0.00061)	−0.0001	0.0023
**Physical squared**	−0.00004***	(0.00001)	−0.0001	−0.00002					−0.00004**	(0.00002)	−0.0001	−0.00001
**Vitality squared**	-	-	-	-					−0.00005*	(0.00003)	−0.0001	0.000003
**Emotions squared**	−0.00004***	(0.00002)	−0.0001	−0.00001					−0.00006**	(0.00003)	−0.0001	−0.00001
**Constant**	−0.09898	(0.06297)	−0.2178	0.0296					−0.22122***	(0.07169)	−0.3604	−0.0790

OLS, ordinary least squares; TPM, two-part model; Model 3 = CFQ-R domains that are statistically significant at the 10% level + statistically significant squared terms.

SE = standard error; ***p < 0.01, **p < 0.05, *p < 0.1.

## Discussion

This study is the first attempt, to our knowledge, to develop a mapping function to estimate EQ-5D preference-based values from a condition-specific measure for patients with CF. The results from this relatively large survey of 401 patients with different levels of disease severity confirmed that EQ-5D preference–based values, or utility values, can be estimated from the CFQ-R using mapping functions. These predicted utility values can be used to inform cost effectiveness models. The study sample included a diverse range of CF severity, as measured by FEV_1_ and observed EQ-5D values, with good sample sizes across FEV_1_ severity categories and close to the full range of theoretical EQ-5D scores represented (1 to −0.35 versus 1 to −0.59). The range of CFQ-R scores was also broad, with means from 21 – 77. This represents a broader and more severe range than that included in the CFQ-R validation (mean range = 51 – 92) [24], but similar to that reported by Bradley et al. (mean range = 25 – 85) [25]. The slight difference in ranges may be due sampling methodology, which allowed completion of the questionnaires in the privacy of the patients’ home rather than on site, and as participants were not recruited through clinics they may also represent a less adherent/controlled group. In addition our sample only included adults (aged 18+), and had a slightly higher proportion of females; age and female gender having both been associated lower (worse heath) scores [24]. As mapping is best supported by datasets with a rectangular distribution to increase the predictive performance of the final algorithm across the entire spectrum of scores, this diversity is likely to have contributed to the consistently strong mapping results seen across regression approaches and item and domain level models.

Assessment of models within sample indicated that the item level models (model 5) outperformed the domain models in terms of predicting the mean, the range, minimising RMSE and levels of agreement with the observed EQ-5D utility scores. However, item models suffered from misspecification and there was evidence of some multicollinearity. Domain level models with squared terms were better specified than the item level models and had no problems with multicollinearity apart from where expected in the squared terms. The domain model with squared terms also performed relatively well within sample in terms of RMSE and ICC. Within sample predictions, the TPM performed marginally better than the OLS or Tobit models in terms of RMSE, ICC and the range of predictions.

In the out-of-sample validation, testing of the best performing domain (model 3) and item level models (model 5 or 8) showed that unlike within sample, domain level models performed better in terms of predicting the mean, minimising the RMSE and level of agreement between observed and predicted scores based on ICC, while item level models performed better in terms of predicting the range of scores. OLS models were better at predicting the mean and minimising the RMSE while TPM models tended to have larger RMSEs. The TPM models performed better in terms of ICC with slightly higher ICCs in TPM model 3 compared to the same model in OLS. Overall, the best performing models in out-of-sample validation were the OLS and TPM domain models (model 3); these included the ‘physical functioning’, ‘role functioning’, ‘emotion’, ‘vitality’, ‘eat’, ‘weight’, and ‘digestion’ domains. Thus, given the misspecification and multicollinearity problem associated with item level models, these two domain models are recommended for generating EQ-5D utility scores from CFQ-R data when no utility data exists. These domain model algorithms can be applied to item level data when domain scores are generated, or when item level data is not available as is often the case when effectiveness information is drawn from published trial data.

When considering the ranges of the predicted values of all mapping functions to the observed range of EQ-5D values, there was a tendency of over prediction in all models for observed values of EQ-5D lower than 0.3, and to a lesser extent, under prediction above observed EQ-5D values > 0.9 both within sample and in out-of-sample validation. Over prediction of low preference-based values is not uncommon in the mapping literature when mapping to the EQ-5D [13,26,27]. The sample did not cover the full range of EQ-5D scores and only a small proportion (3%) had scores less than 0, which makes it difficult to accurately predict in this part of the scale. However, as the over prediction occurs at the very severe end of the EQ-5D spectrum, lower than the observed EQ-5D mean reported in the self-reported FEV_1_ ‘severe’ group, this is likely to have limited impact on the application of these algorithms. It is important that the uncertainty around mapped estimates should be considered when applying these values to cost-effectiveness analysis.

It is interesting to note that the respiratory symptoms domain was not a significant predictor of EQ-5D utility in any of the models. This is likely to be due to the fact that the impact of respiratory symptoms is captured through functioning dimensions of the CFQ-R, which map onto the dimensions in the EQ-5D. It is not uncommon for symptoms that are very specific to a condition to be unrelated to utility scores. However, given the focus of respiratory symptoms in CF trials it may be worth exploring the potential to increase sensitivity in utility scores by developing a condition-specific preference-based measure.

### Limitations

Recruitment was conducted through the CF Trust rather than clinical sites; thus diagnosis and FEV_1_ values were self-reported. However this method allowed for the recruitment of a diverse range of participants, with good age and gender variability across severity levels, and FEV_1_ values in line with a CF population [28,29]. Furthermore, the key measures included in the present study were the EQ-5D and the CFQ-R, these are developed to be patient-reported, and the values reported in the present study are in line with those previously reported in CF [25]. A second limitation is the use of a split-sample method for the estimation and validation of the best fitting model. Validation of the model should be conducted on an independent sample rather than a subset as required here due to sample size. However, the cross-validation method employed in this study permitted the best use of the data to maximise the assessment of model performance.

## Conclusion

The modelling approaches applied in this study demonstrated that mapping functions can be applied to CFQ-R datasets to estimate EQ-5D utility values for economic evaluations of interventions for patients with cystic fibrosis where EQ-5D data is not available. However when applying these mapped estimates to cost-effectiveness analysis, the uncertainty around the extremes of the EQ-5D spectrum should be considered. In addition, further research around the performance of the model in an independent sample is recommended. Finally, given the fact the respiratory domain was not a significant predictor of EQ-5D utility, and the emphasis on respiratory symptoms in CF trials, the development of a disease specific preference based measure may also be worth further investigation.

## Additional file

Additional file 1:
**Mapping the EQ-5D index from the Cystic Fibrosis Questionnaire-Revised using multiple modelling approaches.** Summary of Observed and Predicted Values by EQ-5D group and FEV_1_ Severity.
