# Supplementary material for: Mapping the EQ-5D index from the cystic fibrosis questionnaire-revised using multiple modelling approaches
Source: Health Qual Life Outcomes. 2015 Mar 12;13:33. doi: 10.1186/s12955-015-0224-6 (PMC4364087; doi:10.1186/s12955-015-0224-6)
Supplement: Additional file 1: — Mapping the EQ-5D index from the Cystic Fibrosis Questionnaire-Revised using multiple modelling approaches. Summary of Observed and Predicted Values by EQ-5D group and FEV1 Severity. [file 12955_2015_224_MOESM1_ESM.docx]

Additional file 1: Mapping the EQ-5D index from the Cystic Fibrosis Questionnaire-Revised using multiple modelling approaches

*Table S1: Summary of Observed and Predicted Values by EQ-5D group and FEV_1_ Severity – Cross Validation Sample 1*

|  | **EQ-5D** | **OLS model 3** | **RMSE** | **OLS model 5** | **RMSE** | **Tobit model 3** | **RMSE** | **Tobit model 5** | **RMSE** | **TPM model 3** | **RMSE** | **TPM model 8** | **RMSE** |
| --- | --- | --- | --- | --- | --- | --- | --- | --- | --- | --- | --- | --- | --- |
| **EQ-5D range** |  |  |  |  |  |  |  |  |  |  |  |  |  |
| **-0.349-0.099** | -0.019 | 0.375 | 0.394 | 0.440 | 0.459 | 0.362 | 0.454 | 0.436 | 0.454 | 0.367 | 0.386 | 0.453 | 0.472 |
| **0.1 - 0.299** | 0.213 | 0.451 | 0.238 | 0.361 | 0.154 | 0.441 | 0.167 | 0.371 | 0.167 | 0.452 | 0.240 | 0.331 | 0.150 |
| **0.3 - 0.599** | 0.540 | 0.514 | 0.129 | 0.694 | 0.161 | 0.519 | 0.161 | 0.701 | 0.161 | 0.546 | 0.131 | 0.695 | 0.192 |
| **0.6 - 0.699** | 0.671 | 0.626 | 0.097 | 0.620 | 0.123 | 0.628 | 0.143 | 0.626 | 0.143 | 0.650 | 0.108 | 0.658 | 0.200 |
| **0.7 - 0.799** | 0.748 | 0.719 | 0.137 | 0.709 | 0.180 | 0.707 | 0.186 | 0.704 | 0.186 | 0.738 | 0.144 | 0.672 | 0.206 |
| **0.8 - 0.899** | 0.836 | 0.769 | 0.116 | 0.786 | 0.151 | 0.758 | 0.164 | 0.762 | 0.164 | 0.790 | 0.100 | 0.734 | 0.204 |
| **0.9 - 1** | 1 | 0.901 | 0.103 | 0.963 | 0.133 | 0.904 | 0.073 | 0.927 | 0.073 | 0.917 | 0.083 | 0.917 | 0.083 |
|  |  |  |  |  |  |  |  |  |  |  |  |  |  |
| **FEV severity range** |  |  |  |  |  |  |  |  |  |  |  |  |  |
| **< 41% Severe** | 0.545 | 0.520 | 0.205 | 0.535 | 0.179 | 0.513 | 0.179 | 0.538 | 0.179 | 0.524 | 0.192 | 0.521 | 0.202 |
| **41-70% Moderate** | 0.708 | 0.706 | 0.122 | 0.722 | 0.185 | 0.701 | 0.183 | 0.709 | 0.183 | 0.721 | 0.126 | 0.697 | 0.227 |
| **71-100% Mild** | 0.848 | 0.818 | 0.102 | 0.867 | 0.133 | 0.814 | 0.106 | 0.838 | 0.106 | 0.847 | 0.094 | 0.826 | 0.123 |

OLS, ordinary least squares; TPM, two-part model; FEV_1_, percentage of predicted Forced Expiratory Volume in 1 second;

Model 3 = CFQ-R domains that are statistically significant at the 10% level + statistically significant squared terms; Model 5 = All CFQ-R items excluding the health domain items; TPM Model 8 = All CFQ-R items excluding the health domain items + age and gender

*Table S2: Summary of Observed and Predicted Values by EQ-5D group and FEV_1_ Severity – Cross Validation Sample 2*

|  | **EQ-5D** | **OLS model 3** | **RMSE** | **OLS model 5** | **RMSE** | **Tobit model 3** | **RMSE** | **Tobit model 5** | **RMSE** | **TPM model 3** | **RMSE** | **TPM model 8** | **RMSE** |
| --- | --- | --- | --- | --- | --- | --- | --- | --- | --- | --- | --- | --- | --- |
| **EQ-5D range** |  |  |  |  |  |  |  |  |  |  |  |  |  |
| **-0.349-0.099** | -0.044 | 0.258 | 0.302 | 0.203 | 0.247 | 0.259 | 0.224 | 0.180 | 0.224 | 0.219 | 0.287 | 0.103 | 0.228 |
| **0.1 - 0.299** | 0.228 | 0.378 | 0.185 | 0.322 | 0.271 | 0.371 | 0.266 | 0.342 | 0.266 | 0.352 | 0.185 | 0.291 | 0.237 |
| **0.3 - 0.599** | 0.457 | 0.489 | 0.118 | 0.483 | 0.170 | 0.483 | 0.185 | 0.491 | 0.185 | 0.509 | 0.123 | 0.543 | 0.222 |
| **0.6 - 0.699** | 0.659 | 0.632 | 0.101 | 0.710 | 0.163 | 0.630 | 0.171 | 0.710 | 0.171 | 0.668 | 0.115 | 0.734 | 0.200 |
| **0.7 - 0.799** | 0.759 | 0.744 | 0.094 | 0.762 | 0.145 | 0.749 | 0.148 | 0.748 | 0.148 | 0.776 | 0.110 | 0.834 | 0.159 |
| **0.8 - 0.899** | 0.839 | 0.804 | 0.103 | 0.726 | 0.157 | 0.799 | 0.174 | 0.745 | 0.174 | 0.819 | 0.104 | 0.694 | 0.213 |
| **0.9 - 1** | 1 | 0.920 | 0.080 | 0.942 | 0.109 | 0.918 | 0.067 | 0.933 | 0.067 | 0.938 | 0.062 | 0.909 | 0.091 |
|  |  |  |  |  |  |  |  |  |  |  |  |  |  |
| **FEV severity range** |  |  |  |  |  |  |  |  |  |  |  |  |  |
| **< 41% Severe** | 0.560 | 0.550 | 0.141 | 0.495 | 0.200 | 0.543 | 0.209 | 0.506 | 0.209 | 0.564 | 0.143 | 0.546 | 0.207 |
| **41-70% Moderate** | 0.681 | 0.687 | 0.102 | 0.735 | 0.142 | 0.687 | 0.135 | 0.724 | 0.135 | 0.699 | 0.112 | 0.720 | 0.167 |
| **71-100% Mild** | 0.694 | 0.754 | 0.131 | 0.777 | 0.171 | 0.756 | 0.168 | 0.784 | 0.168 | 0.778 | 0.132 | 0.779 | 0.160 |

OLS, ordinary least squares; TPM, two-part model; FEV_1_, percentage of predicted Forced Expiratory Volume in 1 second;

Model 3 = CFQ-R domains that are statistically significant at the 10% level + statistically significant squared terms; Model 5 = All CFQ-R items excluding the health domain items; TPM Model 8 = All CFQ-R items excluding the health domain items + age and gender

*Table S3: Summary of Observed and Predicted Values by EQ-5D group and FEV_1_ Severity – Cross Validation Sample 3*

|  | **EQ-5D** | **OLS model 3** | **RMSE** | **OLS model 5** | **RMSE** | **Tobit model 3** | **RMSE** | **Tobit model 5** | **RMSE** | **TPM model 3** | **RMSE** | **TPM model 8** | **RMSE** |
| --- | --- | --- | --- | --- | --- | --- | --- | --- | --- | --- | --- | --- | --- |
| **EQ-5D range** |  |  |  |  |  |  |  |  |  |  |  |  |  |
| **-0.349-0.099** | 0.013 | 0.333 | 0.320 | 0.454 | 0.441 | 0.329 | 0.530 | 0.543 | 0.530 | 0.345 | 0.332 | 0.537 | 0.485 |
| **0.1 - 0.299** | 0.225 | 0.469 | 0.244 | 0.545 | 0.320 | 0.466 | 0.312 | 0.537 | 0.312 | 0.475 | 0.250 | 0.522 | 0.312 |
| **0.3 - 0.599** | 0.521 | 0.567 | 0.168 | 0.580 | 0.188 | 0.561 | 0.203 | 0.576 | 0.203 | 0.586 | 0.175 | 0.574 | 0.248 |
| **0.6 - 0.699** | 0.655 | 0.606 | 0.121 | 0.627 | 0.159 | 0.610 | 0.164 | 0.621 | 0.164 | 0.632 | 0.120 | 0.647 | 0.206 |
| **0.7 - 0.799** | 0.764 | 0.776 | 0.069 | 0.719 | 0.123 | 0.790 | 0.157 | 0.709 | 0.157 | 0.800 | 0.091 | 0.757 | 0.170 |
| **0.8 - 0.899** | 0.839 | 0.751 | 0.115 | 0.739 | 0.176 | 0.771 | 0.200 | 0.726 | 0.200 | 0.797 | 0.116 | 0.736 | 0.205 |
| **0.9 - 1** | 1 | 0.842 | 0.158 | 0.846 | 0.186 | 0.858 | 0.140 | 0.861 | 0.140 | 0.872 | 0.128 | 0.823 | 0.177 |
|  |  |  |  |  |  |  |  |  |  |  |  |  |  |
| **FEV severity range** |  |  |  |  |  |  |  |  |  |  |  |  |  |
| **< 41% Severe** | 0.496 | 0.562 | 0.171 | 0.604 | 0.256 | 0.567 | 0.255 | 0.596 | 0.255 | 0.584 | 0.174 | 0.627 | 0.314 |
| **41-70% Moderate** | 0.781 | 0.707 | 0.160 | 0.688 | 0.191 | 0.712 | 0.194 | 0.676 | 0.194 | 0.734 | 0.149 | 0.689 | 0.223 |
| **71-100% Mild** | 0.769 | 0.770 | 0.096 | 0.781 | 0.144 | 0.782 | 0.144 | 0.783 | 0.144 | 0.806 | 0.107 | 0.787 | 0.146 |

OLS, ordinary least squares; TPM, two-part model; FEV_1_, percentage of predicted Forced Expiratory Volume in 1 second;

Model 3 = CFQ-R domains that are statistically significant at the 10% level + statistically significant squared terms; Model 5 = All CFQ-R items excluding the health domain items; TPM Model 8 = All CFQ-R items excluding the health domain items + age and gender

*Table S4: Summary of Observed and Predicted Values by EQ-5D group and FEV_1_ Severity – Cross Validation Sample 4*

|  | **EQ-5D** | **OLS model 3** | **RMSE** | **OLS model 5** | **RMSE** | **Tobit model 3** | **RMSE** | **Tobit model 5** | **RMSE** | **TPM model 3** | **RMSE** | **TPM model 5** | **RMSE** |
| --- | --- | --- | --- | --- | --- | --- | --- | --- | --- | --- | --- | --- | --- |
| **EQ-5D range** |  |  |  |  |  |  |  |  |  |  |  |  |  |
| **-0.349-0.099** | -0.059 | 0.254 | 0.313 | 0.295 | 0.354 | 0.255 | 0.342 | 0.283 | 0.342 | 0.229 | 0.287 | 0.382 | 0.441 |
| **0.1 - 0.299** | 0.239 | 0.414 | 0.199 | 0.358 | 0.260 | 0.423 | 0.283 | 0.343 | 0.283 | 0.422 | 0.204 | 0.561 | 0.416 |
| **0.3 - 0.599** | 0.531 | 0.469 | 0.123 | 0.412 | 0.140 | 0.463 | 0.189 | 0.391 | 0.189 | 0.476 | 0.125 | 0.372 | 0.182 |
| **0.6 - 0.699** | 0.654 | 0.614 | 0.114 | 0.640 | 0.188 | 0.614 | 0.211 | 0.620 | 0.211 | 0.643 | 0.126 | 0.610 | 0.169 |
| **0.7 - 0.799** | 0.753 | 0.738 | 0.051 | 0.715 | 0.147 | 0.743 | 0.168 | 0.721 | 0.168 | 0.779 | 0.070 | 0.729 | 0.202 |
| **0.8 - 0.899** | 0.848 | 0.840 | 0.112 | 0.840 | 0.174 | 0.831 | 0.136 | 0.808 | 0.136 | 0.844 | 0.095 | 0.769 | 0.177 |
| **0.9 - 1** | 1 | 0.913 | 0.091 | 0.854 | 0.155 | 0.913 | 0.076 | 0.924 | 0.076 | 0.935 | 0.066 | 0.896 | 0.104 |
|  |  |  |  |  |  |  |  |  |  |  |  |  |  |
| **FEV severity range** |  |  |  |  |  |  |  |  |  |  |  |  |  |
| **< 41% Severe** | 0.636 | 0.574 | 0.132 | 0.606 | 0.175 | 0.574 | 0.184 | 0.623 | 0.184 | 0.590 | 0.126 | 0.579 | 0.208 |
| **41-70% Moderate** | 0.635 | 0.644 | 0.108 | 0.642 | 0.178 | 0.644 | 0.179 | 0.633 | 0.179 | 0.666 | 0.112 | 0.644 | 0.197 |
| **71-100% Mild** | 0.693 | 0.714 | 0.127 | 0.664 | 0.189 | 0.718 | 0.162 | 0.682 | 0.162 | 0.734 | 0.117 | 0.723 | 0.215 |

OLS, ordinary least squares; TPM, two-part model; FEV_1_, percentage of predicted Forced Expiratory Volume in 1 second;

Model 3 = CFQ-R domains that are statistically significant at the 10% level + statistically significant squared terms; Model 5 = All CFQ-R items excluding the health domain items; TPM Model 8 = All CFQ-R items excluding the health domain items + age and gender
